# The Occurrence of Glycosylated Aroma Precursors in *Vitis vinifera* Fruit and *Humulus lupulus* Hop Cones and Their Roles in Wine and Beer Volatile Aroma Production

**DOI:** 10.3390/foods10050935

**Published:** 2021-04-24

**Authors:** Andrew Caffrey, Susan E. Ebeler

**Affiliations:** 1 Department of Viticulture and Enology, University of California, Davis, CA 95616, USA; ajcaffrey@ucdavis.edu; 2 Food Safety and Measurement Facility, University of California, Davis, CA 95616, USA

**Keywords:** glycosides, grapes, hops, analysis, aroma, fermentation, brewing, winemaking

## Abstract

Volatile aroma compounds found in grapes and hops may be present as both free volatiles and bound glycosides. Glycosides found in the raw materials are transferred to their respective fermented beverages during production where the odorless compounds may act as a reservoir of free volatiles that may be perceived by the consumer if hydrolyzed. A review of the literature on grape and wine glycosides and the emerging literature for glycosides in hops is presented in order to demonstrate the depth of history in grape glycoside research and may help direct new research on hop glycosides. Focus is brought to the presence of glycosides in the raw materials, the effect that winemaking and brewing have on glycoside levels, and current methods for the analysis of glycosidically linked aroma compounds.

## 1. Occurrence of Glycosides in Plant Materials

Glycosidically bound aroma molecules are common secondary metabolites that have been identified in dozens of different plant families [1]. Genotypic evidence has shown that the family of enzymes responsible for glycosylation (UDP glycotransferases) had developed within plants during the transition from water-bound algae to land-based vascular organisms [2]. UDP glycotransferase (UGT) enzymes provide the primary mechanisms for plants to transfer sugar moieties to various classes of molecules such as nucleic acids, proteins, lipids, polyphenols, and various small molecules [3]. Reports of the identification of the first glycosidically bound aroma molecules date back to the work of Francis and Allcock in 1969 where glycosides were isolated from rose petals and hydrolyzed in acid conditions to reveal geraniol and other monoterpene alcohols [4].

Glycosides have been identified in almost every type of plant tissue including leaves, roots, stems, and reproductive organs (i.e., flowering and fruiting bodies) [1]. The direct role of glycosides in plants is still uncertain, but it is hypothesized that glycosides were a part of the “chemodiversity” necessary for plants to survive [2]. The addition of sugar moieties onto hydrophobic aglycones changes the overall polarity and water solubility of the aglycone. The increased polarity of the glycoside allows for detoxification, storage, and transport within the plant [5,6,7,8]. For example, it was shown that the glycosylation of small hydrophobic molecules prevents the disruption of plasma membranes and formation of leaky cell membranes [7,9]. Grapes provide an example where cells exposed to smoke glycosylate the exogenous smoke taint volatiles as a potential detoxification mechanism. This same glycosylation pattern has also been observed in other plants such as *Fragaria x ananassa* (strawberries) [10,11,12]. It is further hypothesized that reservoirs of glycosides could be hydrolyzed for plant defense against microbes and herbivores, plant–plant communications, and as signals to beneficial organisms such as seed disseminators [7,8,13]. In addition, glycosylation enzymes have been shown to be up-regulated in tobacco plants where cells have undergone damage [14].

Plant UGTs are believed to be cytoplasmic enzymes where both endogenous and exogenous small molecules may be glycosylated [15]. After glycosylation, glycosides may be transferred to the vacuole to act as a reserve [16]. When needed, plants hydrolyze aroma glycosides, such as monoterpenyl glycosides, in the tonoplast of the cell [17,18]. When plant materials are harvested, there will be some portion of molecules still stored as glycosides. These have the potential to impact the chemical and sensory profiles of products made from these materials [1].

## 2. Glycosides Found in Grapes and Hops

Through their association with aroma molecule aglycones, glycosides contribute to the aroma profile of fermented beverages such as wine and beer. Several cultivars of *Vitis vinifera* and *Humulus lupulus* have extensive glycosylation patterns that allow for the accumulation of glycosides in the plant tissues that can be released in their respective beverage products [19,20]. *V. vinifera* berries and *H. lupulus* hop cones contain their own unique glycotransferases within the VvGT and HlUGT protein families [21,22,23,24]. Glycosylation patterns and the role of glycosylated compounds on the sensory properties of beverages made with *V. vinifera* berries have been investigated over the last few decades, while research into *H. lupulus* glycosides and their effects on beer aroma and flavor is an emerging field [1,20,25]. The extent that glycosides affect wine aroma is still being investigated, but there is evidence that glycosides act as a reservoir for aroma compounds as they are hydrolyzed by enzymes or by acid-catalysis mechanisms during the fermentation and ageing process [26,27,28,29].

Grapes and hops have been found to contain several classes of volatile molecule aglycones that include: monoterpene alcohols, monoterpene polyols, norisoprenoids, sesquiterpenoids (not reported in hops), aliphatic alcohols, and volatile phenols [30,31,32,33]. A large breadth of research on aroma glycosides in plant materials has largely focused on grapes. There have been numerous studies on the impacts of external factors (i.e., growing and harvest year, ripening events, and vineyard practices) on grape glycosides [24,34,35,36,37]. For example, vineyard practices such as leaf removal as a part of canopy management have shown to increase the abundances of aroma glycosides in Riesling and Chardonnay varieties [36,37]. In addition to endogenous grape metabolites, it is also known that grapes can take in exogenous volatiles, such as volatile phenols produced during wildfires, and convert them to glycosides within the berry [10,11,12].

The extent of investigations into the glycoside composition of hop cones has focused largely on monoterpene alcohols, compounds often seen as driving factors for the aroma of several hop varieties, and norisoprenoids, such as β-damascenone [20,38,39,40]. However, less information into the development of these glycosides in hops is known, when compared to grapes, although it does appear that external factors such as soil, climate, and agricultural practices can influence glycoside abundances in hop plant materials [33]. It is possible that hops may undergo similar exogenous volatile uptake and glycosylation as grapes and other plants do, but future studies will be needed to investigate these ideas.

While there are several different aglycone classes found in grape and hops glycosides, the glycosylation pattern of glycosides follows specific patterns. In general, the first sugar bound to an aglycone by a UGT enzyme is a glucopyranose [1,22,41,42]. It was generally believed that a glucopyranose sugar was always the primary sugar bound to the aglycone; however, recent tentative identification of sesquiterpene glycosides in Muscat of Alexandria grapes and smoke taint glycosides in Cabernet Sauvignon grapes has shown that it is possible for non-glucosyl sugars to be bound directly to the aglycone [31,43]. Within the glycosylation process, up to three sugars in the glycone have been reported for grapes [35]. Although, there has not been full characterization of the glycone content of hop glycosides, evidence from hydrolysis studies suggests that hop glycosides may have one or two sugars in the glycone [20]. The potential sugars that may be found in the glycone are reported as glucopyranose, apiofuranose, xylopyranose, arabinofuranose, and rhamnopyranose sugars, in addition to malonylated glucosides [1,23,35,44,45]. The release of bound aglycones through enzyme hydrolysis is largely dependent on the sugars present in the glycoside, since specific enzymes are needed to cleave off each terminal sugar before the aglycone is released [1,44,45,46]. Some of these enzymes may not be present during winemaking or brewing, leaving glycosides with specific sugars intact [47,48]. Further discussion of these hydrolysis reactions in winemaking and brewing is included below.

## 3. Glycosides during Winemaking and Brewing

Both winemaking and brewing processes have several production steps that may influence the glycosidic profile in the finished beverage. Within this section, winemaking and brewing processes are discussed in separate sections with emphasis on the process itself in regard to glycosides. Microbial considerations for the winemaking and brewing sections are limited to *Saccharomyces* yeast as they are used for the majority of wine and beer fermentations. The production of wine and beer products with non-*Saccharomyces* microbes has been organized into its own section due to the large scope of yeast and bacteria that can be introduced into grape juice or wort systems. Outlines of winemaking and brewing processes with respect to glycoside hydrolysis may be found in Table 1.

### 3.1. Winemaking

The factors that contribute to glycoside hydrolysis during winemaking are part of a complex system that includes endogenous grape enzymes, microbial enzymes, and a low pH. Hydrolytic enzymes found naturally within the berries are largely ineffective towards the hydrolysis of aroma glycosides after grape processing [45]. Evidence has shown that endogenous grape glycosidase enzymes are largely inhibited by a combination of factors including protein inhibitors, inhibition from sugars such as glucose and fructose, tight ionic associations of the enzymes with cell wall materials, and the low pH of the resulting grape juice [45,49,50,51,52,53]. Ineffective endogenous grape enzymes would leave a large reserve of glycosides for acid and enzyme hydrolysis during fermentation and ageing.

Fermentations also offer the opportunity for microbial glycosidase enzymes to be released into grape must where there is a large potential for hydrolysis to occur during fermentation. The hydrolytic capabilities of *Saccharomyces* strains have been widely researched in the literature, but there is no definitive answer as to the extent that *Saccharomyces* yeasts may contribute to the hydrolysis of glycosides in winemaking. Surveys of oenological *Saccharomyces* glycosidases indicated that the enzymes can hydrolyze glycosidic linkages in model juice and some grape juice systems [43,44,45,46,54,55,56,57,58]. The glycosidases produced from *Saccharomyces* are largely extracellular cell wall modification enzymes [54,59]. Fermentations with oenological yeast genetically modified to overexpress the *EXG1* gene associated with hydrolysis resulted in higher levels of free terpenes when compared to the control in both model solutions and grape must [87]. However, while the glycosidases produced by *Saccharomyces* have been shown to be capable of hydrolysis, the extent that different yeast strains are able to affect the pool of glycosides is unclear as only 1 of 153 different *Saccharomyces cerevisiae* strains was shown to have any recognizable β-glucosidase activity in model systems [60]. Due to inconsistencies among the literature studies on glycosidase activity in oenological yeast, some attention has been brought to alternative enzymes, such as oxidases present in the periplasmic space of *Saccharomyces* yeast, which have been shown to have glycoside hydrolyzing capacities [61]. Still, despite low reported β-glucosidase activities, yeasts are consistently reported to have an influence on the overall abundances of aroma-related glycosides in wine [43,58,62,63].

Glycoside abundances due to yeast glycosidase activity have been shown to have the greatest changes during the active growth phases of fermentation [43,64]. However, it has been reported that some microbial hydrolytic activity may extend beyond the initial growth stages of fermentation [10]. Levels of aroma glycosides were decreased as a direct result of similar secondary fermentation events used to make sparkling Riesling and Chardonnay wines [58].

Investigations into monoterpenol glycosides have revealed that free monoterpenols in finished wines may not be fully derived from grape precursors as some studies have shown that oenological *Saccharomyces* strains were able to influence the overall monoterpenol profile without affecting the abundances of total glycosides [27,29,54,58]. The possible de novo synthesis of monoterpenols by yeast may be considered a case where monoterpenol content was able to increase without direct decreases in glycoside abundances. Under high growth and low oxygen conditions, geranyl diphosphate may be a by-product of the mevalonate pathway in *Saccharomyces cerevisiae* [88,89,90]. The mevalonate pathway is seen to be upregulated in growing yeasts as ethanol-induced changes in membrane fluidity increase demand for sterols [91,92,93,94].

Investigations into the release of aglycones through the secondary fermentation of sparkling wines found that trends in free volatile content did not always correlate to direct additions of glycosylated aroma precursors to the wines. The reason for a lack of correlation between exogenous glycoside additions and the free volatiles were attributed to complex interactions with the wine matrix and biotransformation of the free volatiles during the fermentation process [58]. Because of the complexities and different metabolic sources of free volatiles in wines, additional studies monitoring the abundances of glycosides throughout fermentation are necessary for deeper insights into the biochemical pathways associated with glycoside-related aroma compounds beyond hydrolysis.

### 3.2. Brewing

Traditionally, hop glycosides were perceived to have minimal impact on the aroma profile of beer; however, changes in consumer preferences towards citrus and tropical aromas has increased the dosage rate of hop material in beers [95,96]. Additionally, dry hopping beers can lead to a phenomenon called hop creep where endogenous hop enzymes act on residual sugars in finished beers and retrigger fermentation [85,86]. Monoterpene levels in hops have been shown to be impacted by complex interactions with several facets of influx and outflux, with oxidation, hydrolysis, and biotransformation being factors [95,97].

Hydrolysis of hops glycosides has been observed in beer production. Increases in both linalool and β-damascenone have been reported during the boiling and fermentation of wort containing Saaz, Tettnang, and Hersbrucker hops [40]. However, it takes large amounts of hop materials (amounts greater than 10 g hops per liter of wort) to have monoterpenol glycosides make a noticeable effect on the final sensory aroma of the finished beers [20,83]. To combat this high usage rate, methods to extract glycosides from hops to use as beer flavorants have been explored [98]. Since hops may be added at several stages in the brewing process, the timing of hop additions and the timeline of hydrolytic events, including fermentation, are important considerations for the contribution of glycosides on the final aroma of beer [10,43,95].

In modern day brewing practices, hops may be added at several places throughout the brewing process. Hops are traditionally added during the boiling stage, but hops can also be added at the conclusion of the boil, during the whirlpool stage, and before, during, or after fermentation. There is a lack of information about how glycosides may behave when hops are added at each of the previously listed times in the brewing process. Each stage offers its own unique conditions that could affect glycosidic content differently. For example, glycosides present during the boiling process of wort experience an acidic solution (generally a pH of 5.1–5.4) and are held at boiling temperatures for prolonged periods of time ranging from as low as 45 min to upwards of 90 min. Hop-derived isoprenoids, most notably β-damascenone, have been shown to increase during the boiling stage of the brewing process [40]. A separate study that screened several hop varieties for glycosides using both enzyme and acid hydrolysis methods found that β-damascenone was an exclusive product of glycoside hydrolysis only under acidic conditions [20]. The high temperatures and acidic conditions may promote the extraction and hydrolysis of aroma glycosides from hop materials; however, the volatilization of aromas during the boil will likely minimize the impact of volatiles released during this stage, as free monoterpenols are shown to be lost during the boiling process [40].

Although hops added during the boiling process may not directly impact beers in the forms of glycosides, hop additions that extend beyond the boiling stage of brewing have a potential for glycoside-related impact. Once the boiling of wort has concluded, it is still possible to add hops during the whirlpool stage, as well as before, during, or after the primary fermentation with *Saccharomyces* yeast. The timing of hop additions is important with respect to hop-derived compounds, such as monoterpenes, as they interact with several metabolic pathways of *Saccharomyces* yeast [95]. There is a potential for the loss of liberated aglycones if glycosides are hydrolyzed too early in the fermentation or final production processes. Events such as fermentation and the use of carbonation stones in beers generate carbon dioxide bubbles that have been shown to interact with free volatiles in matrices such as beer and wine, pulling them out of solution and volatilizing them into the headspace when the bubbles reach the surface of the liquid [99]. Direct evidence has shown that common aglycones that are released by the hydrolysis reactions, e.g., linalool, are lost due to carbon dioxide generation in both beer and wine fermentations [100,101,102,103]. However, it is important to keep in consideration that glycosides must be hydrolyzed before the free volatiles can leave the solution.

A screening of 58 *Saccharomyces* brewing strains showed that the yeasts have minimal β-D-glucosidase activity during beer fermentation, but some strains expressed exo-1,3-β-glucanase activity that has the capability to hydrolyze glycosides [65]. Kanauchi and Bamforth [84] later reinforced the results from Daenen et al. [65] by finding that brewing strains had minimal capabilities to hydrolyze glycosidic linkages with exogenous glucosidases. Despite indications of minimal glucosidase activity, Sharp et al. [83] found that ale yeasts were able to induce hydrolysis of glycoside inoculated wort. In this study, two ale yeasts were chosen, one reportedly having a high glucosidase activity and the other minimal glucosidase activity. Both strains exhibited the same level of glycoside hydrolysis during wort fermentations, demonstrating that other factors, such as exo-1,3-β-glucanase activity, may be at play [65,83]. Much like in wine, the hydrolytic ability of brewing strains is unclear and more studies are needed to understand the complex interaction between yeast and aroma glycosides during fermentation.

Even if glycosides are hydrolyzed during fermentation and the released aglycones are not lost due to volatilization, the presence of free aglycones during active parts of fermentation may have unpredictable effects on the flavor outcome, as some hop-derived monoterpenols are subject to biotransformation, e.g., transformation of geraniol to citronellol [95,97,104,105]. Furthermore, if glycosides are not hydrolyzed by yeast during fermentation, the aglycones may be still be spontaneously released by acid hydrolysis during aging, depending on the beer production process. In general, acid hydrolysis of glycosides that remain in the beer is generally not considered to impact the final product as most beers are not aged due to the undesirable formation of trans-2-nonenol during the ageing process [106]. However, beer has a multitude of different styles that may be aged. Belgian beers have been shown to develop changes in aroma due to the acid hydrolysis of glycosides during the ageing process [39]. Other emerging beer styles such as “oenobeers”, where wort is fermented with grape material present, pose interesting possible interactions between glycosides found in both grapes and brewing conditions. Evidence has shown that the hydrolysis of glycosides contributes free volatiles to the chemical aroma profiles of wines, but sensory studies on beer are needed to determine the extent that the released aglycones impact the aroma perceived by consumers.

### 3.3. Roles of Non-Saccharomyces Fermentations on Glycoside Hydrolysis

Both beer and wine production have products that may undergo fermentation with non-*Saccharomyces* microbes. Commonly used organisms with observed hydrolytic activity can include yeasts such as *Hanseniaspora* or *Brettanomyces* in addition to lactic acid bacteria such as *Oenococcus* and *Lactobacillus* [65,66,67,68,69,70]. Investigations into the hydrolytic capabilities of non-*Saccharomyces* fermentations show that there is a higher potential for glycoside hydrolysis, but the increased hydrolytic capability accompanies several considerations such as the differences in the nature of the hydrolytic enzymes used by non-*Saccharomyces* organisms and the unpredictable nature of non-*Saccharomyces* microbes. For example, malolactic fermentation in red wines was shown to decrease the total monoterpene glycoside content of red and white wines when compared to controls without always seeing a corresponding effect on the free monoterpene profile [71,72,73].

It is believed that the increased hydrolytic capabilities of different microbes do not always translate to an increase in free volatiles due to the cellular locations of the enzymes [60]. An overwhelming majority of studies cite the location of *Oenococcus oeni* glycosidases as cell membrane proteins or cytoplasmic proteins [70,74,75,76,77,107,108]. Glycosidases from *Oenococcus oeni* have the hydrolytic capacity to release aglycones from each compound class listed by Caffrey et al. [31,78]. However, since the glycosidase enzymes are reported to be intracellular, in order to have an effect on the overall aroma, glycosides must be imported into the cell, hydrolyzed, and have the free volatile diffuse back into the beverage matrix without binding to cell components. Requiring glycosides to enter a cell for hydrolysis is a large obstacle to the release of aglycones as it is hypothesized that glycosides or freed aglycones may bind to mannoproteins or polysaccharides produced during malolactic fermentation, attenuating the impact on the final aroma composition [71,72,77]. Limitations to glycoside hydrolysis by microorganisms extend beyond the cellular location of hydrolytic enzymes as well. The expression of glycosidases is somewhat unpredictable as studies that screened dozens of strains of *Oenococcus oeni* found that factors such as ethanol content, pH, temperature, and residual sugars all impact the hydrolytic capacity of the bacteria in different strain-dependent ways [70,76].

Although the use of non-*Saccharomyces* yeasts is not as prevalent as conventional *Saccharomyces* fermentations, there is increasing interest in “natural” fermentations in the wine and beer industries. The emergence of wines made through spontaneous fermentation with microbes indigenous to the winery have been shown to allow for a higher hydrolytic rates of glycosides compared to fermentations inoculated with industrial strains [69,79,80]. Additionally, producers of certain beer and beer-related products, like Iambics, intentionally inoculate with microbes such as *Brettanomyces* that carry hydrolytic capabilities [65,68]. Although there is a higher capability to release compounds such as monoterpenols in the finished product, the use of alternative yeasts has its own implications. Aroma considerations beyond glycoside precursor hydrolysis need to be accounted for when considering usage of non-*Saccharomyces* yeast and bacteria, since many of these organisms are considered spoilage microbes within conventional wines and beer styles.

## 4. Current Approaches for Glycoside Analysis

Although it has been known for almost four decades that grapes and wines contain aroma-related glycosides, comprehensive studies on their dynamic abundances in fermented products have been limited by analytical technologies [109,110]. Most aroma glycoside classes, such as the isoprenoid-glycosides, do not generally contain chromophores that would allow for their direct detection through high performance liquid chromatography (HPLC) ultraviolet visible light spectroscopy (UV–vis) experiments. Generally speaking, non-volatile glycosides are required to undergo induced hydrolysis through harsh acidic conditions or the addition of exogenous enzymes followed by analysis of the released volatiles with gas chromatography mass spectrometry (GC/MS) in order to characterize the aglycones [111,112]. These strategies, however, are not ideal for the quantitation of aroma molecules since enzymes often have stereoselective preferences based on aglycone identities, and acid hydrolysis causes intramolecular rearrangement [111,113]. The enzymatic mechanisms of glycosidases are more protective of the aglycone structure by allowing for a nucleophilic attack on the anomeric carbon in the sugar, whereas acidic hydrolysis may have a carbocation generation on the aglycone where compounds such as monoterpenols or norisoprenoids will rearrange [81,114,115]. Recent research into the hydrolysis methods for studying hop glycosides reinforces that enzymes show the most promise for characterizing the intact glycosides and the associated aglycones [20].

Often times, studies forgo measuring glycosides and instead analyze the effects of different experimental conditions, such as fermentation temperature or yeast strain selection, on the abundances of free volatiles [63]. The information gained by only measuring the free volatiles can be insightful by highlighting which yeast strain or enzymatic treatments may be effective in attenuating or increasing the hydrolysis of glycosides; however, the picture is incomplete. In the absence of intact glycoside measurements, concentrations of free volatiles at any given time may not reflect future concentrations because acid hydrolysis can occur slowly over time. For example, large reservoirs of aroma precursors are of great concern for smoke-tainted wine as the smoke-taint volatile phenols can be released from glycoside precursors during wine storage [116]. Studies that have used the addition of exogenous enzymes to understand the aroma potential of glycosides present in wines found that glycoside reserves are capable of changing the overall sensory profile of the studied wines [117]. Therefore, it is vital to understand that even though there is a potential for the release of volatiles during fermentation, large portions of glycoside reserves, as high as three quarters of the original total abundance, may remain after fermentation and can alter the wine aroma over time [29,43,82,118]. These large reserves of glycosylated aroma compounds that are capable of hydrolyzing over time have the potential to create a product that is different from that intended by the winemaker. Using smoke taint as an example, it is highly desirable to limit the amount of free volatile phenols in affected wines. Wine treatments such as reverse osmosis, a technique that aims to ameliorate the abundances of detrimental free volatile phenols in wines, can be rendered ineffective due to the reappearance of volatile phenols from the acid hydrolysis of smoke taint glycosides [119].

In the absence of glycoside measurements, aroma molecules bound to glycosides may initially go undetected only to resurface during consumption. For example, glycosides have been found to alter the retronasal aroma of wines through in-mouth hydrolysis within two minutes of ingestion, even though the wines had little glycoside-related orthonasal aroma [120,121]. Because of the potential for glycosides to hydrolyze over time in the bottle or in the mouths of consumers, directly measuring glycosides can help predict the aroma potential of wines. Understanding the future aroma of a wine may allow winemakers to make informed decisions concerning the wine production process and when it is best to consume the wines produced.

Recent advances in ultrahigh performance liquid chromatography mass spectrometry (UHPLC/MS) have allowed for the direct measurement of glycosides. Direct measurements allow for more in-depth and detailed understanding of how glycoside profiles change over time during crop development or winemaking and brewing processes. However, the results are limited in scope due to a lack of analytical standards leaving identification of molecules tentative and determination of concentrations semi-quantitative [31,34,35,43]. Larger limitations for LC/MS analysis are in the identification of aglycones. The highly isomeric nature of plant-derived aroma compounds makes confirmed identifications difficult [31,34,35,122,123,124]. Other methods of separation and isolation are needed to understand the aglycone structure of glycosides. This is typically performed with chromatographic fractionation followed by sequential enzyme hydrolysis or nuclear magnetic resonance spectroscopy [44,125]. Methods for the analysis of aroma glycosides with LC/MS generally use electrospray ionization (ESI) or atmospheric chemical ionization (APCI) [126]. Although there is evidence that both methods are useful in the analysis of glycosides, current trends favor ESI over APCI due to its better sensitivity [43,62]. LC/MS analysis does offer advantages to studying glycosides in comparison to traditional GC/MS methods. GC/MS hydrolysis experiments often rely on more readily available standards, commonly p-nitrophenol glycosides, to indirectly understand hydrolysis kinetics and mechanisms of glycosides [44,112,127]. However, use of compounds such as p-nitrophenol glycosides may be misleading as hydrolysis can be dependent on the identity of the aglycone.

## 5. Conclusions

Glycosylated aroma molecules are common metabolites found in the plant families *Vitis vinifera* and *Humulus lupulus*. The glycosylated molecules are of increasing interest due to their connection with free volatile molecules in wine and beer. Comparatively, there is a rich history of knowledge concerning the presence of glycosides and their respective aromas in grapes and wine, while the field of research is starting to develop for hops and beer. A large focus of winemaking has shown that microbial activity during the fermentation process is a key event in the hydrolysis of glycosides. Although much work has shown that microbial activity can trigger the hydrolytic release of glycosides from wine, there is still more work needed to understand how to control the process. Brewing is a complex process that offers several opportunities for hydrolysis such as the boiling and fermentation stages; however, the impact of glycosides from hops is frequently brought into question due to their low abundances. The development of liquid chromatography mass spectrometry methods has allowed for more in-depth and faster analysis of intact glycosides in both plant materials and fermented beverages when compared to traditional hydrolysis methods. More development is still needed for these methods as it can be difficult to distinguish isomeric aroma glycosides from one another through mass spectrometric methods alone.

## Figures and Tables

**Table 1:** Hydrolytic outline of the winemaking process and the brewing process.

Winemaking Stage	Primary Hydrolysis	References
Destemming and crushing	Endogenous grape enzymes	[45,49,50,51,52,53]
Fermentation—*Saccharomyces*	Exogenous yeast enzymes	[43,44,45,46,54,55,56,57,58,59,60,61,62,63,64]
Fermentation—non-*Saccharomyces* yeast and/or mixed fermentations	Exogenous yeast enzymes	[65,66,67,68,69]
Malolactic fermentation	Exogenous bacterial enzymes	[66,67,70,71,72,73,74,75,76,77,78,79,80]
Racking, bottling, and storage	Spontaneous Acid Hydrolysis	[48,81,82]
**Brewing Stage**	**Primary Hydrolysis**	**References**
Mashing	N/A	N/A
Lautering and sparging	N/A	N/A
Boiling and whirlpool	Acid	[20,40]
Fermentation—*Saccharomyces*	Exogenous yeast enzymes	[65,83,84]
Fermentation—non-*Saccharomyces* yeast and/or mixed fermentations	Exogenous yeast or bacterial enzymes	[65,68]
Dry hopping and hop creep	Exogenous hops enzymes	[85,86]
Storage	Spontaneous Acid Hydrolysis	[39]

## Data Availability

This review does not report any data.
